# Diet of the earliest modern humans in East Asia

**DOI:** 10.3389/fpls.2022.989308

**Published:** 2022-08-31

**Authors:** Yan Wu, Dawei Tao, Xiujie Wu, Wu Liu, Yanjun Cai

**Affiliations:** ^1^Key Laboratory of Vertebrate Evolution and Human Origins, Institute of Vertebrate Paleontology and Paleoanthropology, Chinese Academy of Sciences, Beijing, China; ^2^CAS Center for Excellence in Life and Paleoenvironment, Beijing, China; ^3^Department of Archaeology, School of History, Zhengzhou University, Zhengzhou, China; ^4^Institute of Global Environmental Change, Xi’an Jiaotong University, Xi’an, China

**Keywords:** hominins, diet, starch analysis, dental calculus, plants

## Abstract

Reconstructing diet can offer an improved understanding toward the origin and evolution of modern humans. However, the diet of early modern humans in East Asia is poorly understood. Starch analysis of dental calculus is harmless to precious fossil hominins and provides the most direct evidence of plant food sources in early modern human dietary records. In this paper, we examined the starch grains in dental calculus from Fuyan Cave hominins in Daoxian (South China), which were the earliest modern humans in East Asia. Our results reveal the earliest direct evidence of a hominin diet made of acorns, roots, tubers, grass seeds, and other yet-unidentified plants in marine isotope stage 5 between 120 and 80 ka. Our study also provides the earliest evidence that acorns may have played an important role in subsistence strategies. There may have been a long-lasting tradition of using these plants during the Late Pleistocene in China. Plant foods would have been a plentiful source of carbohydrates that greatly increased energy availability to human tissues with high glucose demands. Our study provides the earliest direct consumption of carbohydrates-rich plant resources from modern humans in China for the first time. In addition, it also helps elucidate the evolutionary advantages of early modern humans in the late Middle and early Upper Pleistocene.

## Introduction

Scientific interest in the origin and evolution of modern humans has a long history. Since the emergence of *Homo erectus*, China has been an important area of human evolution (Gao et al., 2010, 2017). New fossils were found in Huanglong Cave in Hubei (Liu et al., 2009), and at the Zhiren Cave in Guangxi (Liu W. et al., 2010). Moreover, 47 human teeth dating back to more than 80 ka (with an inferred maximum age of 120 ka) by the excavator team were found in Fuyan Cave in Hunan (Liu et al., 2015), which provided the earliest fossil evidence of modern humans in South China. These findings indicated that fossil hominins with full early modern human morphology were present in South and Central China as early as 100 ka (Liu et al., 2016).

Diet is key to understand the origin and evolution of modern humans. Diet is a direct reflection of human adaptation and transformation of the natural environment. Much of human evolutionary success can be attributed to our ability to consume a wide range of foods (Ungar and Teaford, 2002). Reconstructing paleolithic diets can offer an improved perspective on human adaptation and may help elucidate modern human dietary physiology.

Plants are an important dietary source for humans. In the study of the utilization of plant resources, there is not only evidence of macrobotanical remains, but also evidence of microbotanical remains. However, the evidence of macrobotanical remains obtained from the sites are not all related to food, and the plant remains found in dental calculus are more likely to be closely related to human diet. Dental calculus is made of calcium phosphate deposits on teeth that captures many food particles and therefore contains the dietary information of ancient humans (Leonard et al., 2015). Analysis of early Paleolithic dental calculus is particularly valuable (Hardy et al., 2017, 2018); it is both harmless to precious hominin fossils and the data recovered may be the only direct evidence of plant consumption. Starch grains entrapped within the calculus can provide the most direct evidence of items ingested in the past (Hardy et al., 2017).

Recently, DNA and proteomics studies are rising tools for the study of ancient dental calculus, and providing new data for diet (Hardy et al., 2018). However, we have carried out biomolecular analysis on some human dental calculus from Fuyan cave, and unfortunately have not obtained any endogenous ancient DNA data. The recovery of starch grains from dental calculus has become increasingly useful for revealing diets of Neanderthals and early modern humans. On the basis of starch grain analysis, palms, beans, and grasses were found from Neanderthal dental calculus, which revealed a wide spectrum of plant foods sources (Henry et al., 2011). Further analyses of plant derived diets were compared between Neanderthals and other early modern humans from several populations in Europe, the Near East, and Africa (Henry et al., 2014). In East Asia, starch grain analysis studies at Zhuannian (∼10.0 ka), Nanzhuangtou (>11.0 ka), and Shizitan (∼28 ka) confirmed plant foods exploitation strategies by the modern humans (Liu et al., 2013, 2018; Yang et al., 2015). However, these sites are all less than 30,000 years old and located in northern China. Research in East Asia has not been carried out for early modern humans older than 30 ka. Besides, the starch grains recovered from human dental calculus can provide more direct diet evidence than grinding tools (Cristiani et al., 2016). Thus, this paper investigates starch grains on the dental calculus of human teeth dated 120–80 ka from Fuyan Cave in South China to provide important dietary information. This work aims to study the subsistence strategy of early modern humans in early Late Pleistocene and their adaptations to the environment.

## Materials and methods

**FIGURE 1:**
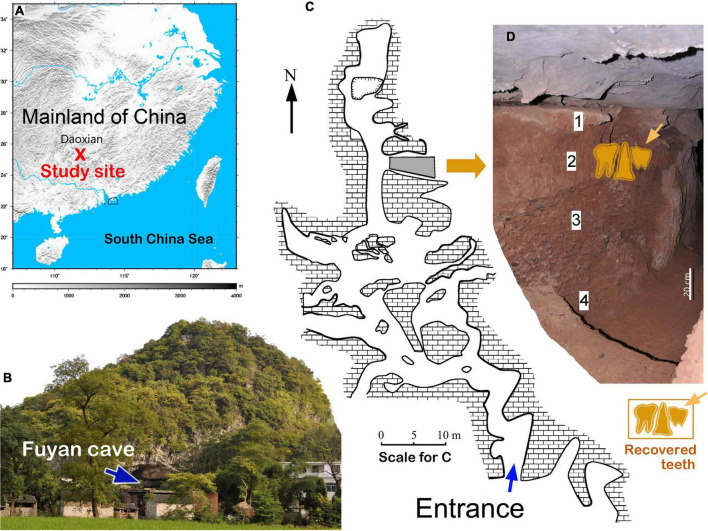
Geographical location and stratigraphy of Fuyan Cave in Daoxian. **(A)** Location of Fuyan Cave. **(B)** Panoramic view of Fuyan Cave. **(C,D)** Stratigraphic layers of region II of Fuyan Cave. All human fossils came from layer Map of **(A)** was generated using GMT 5.2.1. (http://gmt.soest.hawaii.edu/home). **(C)** Modified from Liu et al. (2015).

Fuyan Cave is located in Tangbei Village, Daoxian County, Hunan Province, South China (E 111°28′49.2″, N 25°39′02.7″) (Figure 1A). Fuyan Cave is a karst system that comprises several connected and stacked chambers (Figure 1B), and covers a total area of more than 3,000 m^2^ (Figure 1B). Three seasons of systematic excavation at Fuyan Cave in Daoxian were carried out between 2011 and 2013, and yielded 47 human teeth and an abundant fossil mammalian assemblage (Figures 1C,D). The morphological and metric assessment of those teeth supports their unequivocal assignment to fully modern humans (Liu et al., 2015). The original study used both U-series of the flowstone and the biostratigraphic information to support the 120–80 ka; this is the earliest and soundest evidence of modern humans in South China (Liu et al., 2015).

Recently, Sun et al.’s (2021) study showed new chronologies of Fuyan Cave, suggesting that their chronologies are Holocene. Higham and Douka (2021) and Martinón-Torres et al. (2021) pointed out Sun et al.’s major issue were derived from unreliable “human teeth” and other dating samples collection, “human teeth” identification, problematic dating method. Considering the complexity of cave deposits and cave strata, and the fact that Sun and the initial excavation team did not confirm their samples came from the exact same location and layer (Martinón-Torres et al., 2021; Sun et al., 2021), Sun et al.’s study were irrelevant to our study since their samples are were not the same assemblage as ours, and even not the same excavation area and the same stratum. Recently, the U-series dates of fossil tooth *Bos (Bibos) gaurus* Smith, 1,827 excavated along with the human teeth at the same layer (layer 2) at Fuyuan cave confirms the original age to be older than 80 ka (Cai et al., unpublished data). Based on these reasons, we suggest that the original excavator’s determination of the age for our samples should be more reliable, and therefore, the age determined by the excavation team is applied here in this study.

**FIGURE 2:**
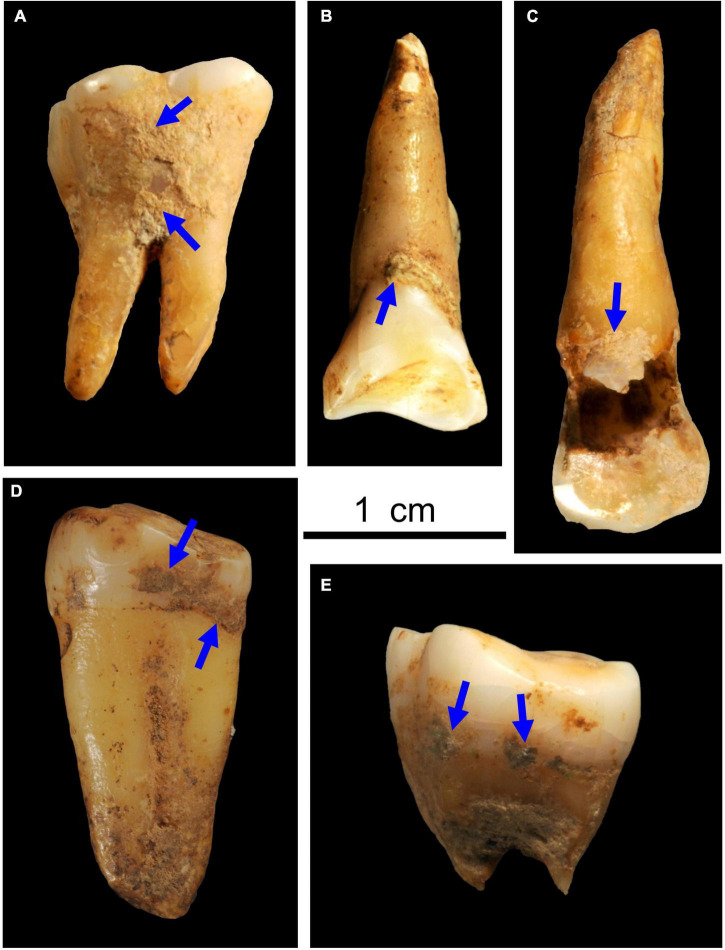
Images showing deposition of dental calculus on Fuyan Cave hominins teeth [Sample No. **(A)** DX4. **(B)** DX.2. **(C)** DX13. **(D)** DX6 **(E)** DX5] (Scale bar = 1 cm).

Adhered soil and other particles on 47 human teeth from Fuyan Cave in Daoxian were cleaned with a soft tooth brush. Microwear and dental calculus were observed under a digital microscope (VHX-600) using the extended depth of focus (EDF) technology. First, surface of teeth was thoroughly cleaned by blower and acetone before extracting dental calculus. Fifteen teeth had obvious calculus (Figure 2), and a dental pick was used to scrape visible calculus following the non-destructive protocols outlined by Piperno et al. (2000) and Henry et al. (2012).

To prevent samples from being contaminated, all implements were cleaned in an ultrasonic water bath before they were used. Two blank slides were placed on the laboratory table to check the air contamination in Ancient Starch lab of Zhengzhou University before the extraction experiment (Piperno and Dillehay, 2008; Li et al., 2010). When the experiment was finished, the mixture of glycerol and ultrapure water (1:1) were used to mount on the two blank slides. Moreover, to check the contamination during the extraction process, two new empty 2 ml centrifuge tubes were used as control samples. Extraction of starch grains from the two control samples and from human dental calculus were followed the same procedure. The mixture of glycerol and ultrapure water (1:1) were used to mount the “residues” extracted from the two controlled samples on the blank slides. Olympus BX53 were used to examine the four slides. No starch grains were found on the four slides, suggesting that no starch contamination existed in the lab environment and during the ancient starch grains extraction process. Additional experiment on samples of surface sediments of animal teeth does not yield starch grains, but found several phytoliths (Supplementary Figure 1).

Calculus fragments were rinsed in distilled water and transferred into centrifuge tubes. 2 ml 2.9 mol/L HCl was then added and the tubes were left for several hours until there were no more bubbles. It should be noted that the dissolution of calculus with EDTA instead of HCl is now the preferred method (Tromp et al., 2017) as it releases more microremains. For the interested readers who would like to perform similar analysis, employing the EDTA method may give similar results in this paper. Then, distilled water was added and the solution was centrifuged at 3,000 rpm for 10 min. The supernatant was removed and the residue at the bottom was retained. This step was repeated for three times. The samples were then mixed with 5% sodium hexametaphosphate for 24 h. After two washes with distilled water, a heavy liquid flotation with a dense solution of CsCl (*d* = 1.8 g/ml) was added to the sample to float the starch grains. After adding the distilled water into the new centrifuge tube and centrifuging it at 3,000 rpm for 10 min, the supernatant was pipetted and removed. This step was repeated a second time, and the remaining residue was kept for microscopic observation. The remaining sample was mounted in 1:2 glycerin/water on a slide and examined under polarized and transmitted light with an Olympus BX53 at 400 × magnification. Each starch grain was photographed, described, and counted, and the entire slide was examined. We observed and analyzed microwear under a digital microscope (VHX-600) using the EDF technology. In order to make a reasonable identification ancient starch grain, we made a one-to-one comparison with modern references native to the study area (Supplementary Figure 2), and certain modern comparative plants commonly recovered from later sites in this region were employed (Supplementary Figure 3). Existing literature on diagnostic criteria for starch grains was also consulted (Piperno et al., 2004; Chandler-Ezell et al., 2006; Piperno and Dillehay, 2008; Ge, 2010; Yang and Jiang, 2010; Tao et al., 2011, 2015; Wan et al., 2012; Yang and Perry, 2013; Wang, 2017).

## Results

A total of thirty-two starch grains were recovered from the dental calculus of thirteen Fuyan Cave hominins teeth. In addition, two wood fragments with characteristic conifer tracheid fibers were identified in the dental calculus of two Fuyan Cave hominins teeth (see Supplementary Table 1 for detailed starch and wood fragments counts). The source of starch granules was detected by comparing the size, shape, presence, and prominence of lamellae; hilum morphology; formation characteristics; cross sections; cracks; and other surface features. Twenty-two starch grains could be assigned to four main types. The others could not be identified because of damage or a lack of diagnostic features. The morphological characteristics of identified starch types and wood fragments are described below.

### Type 1

**FIGURE 3:**
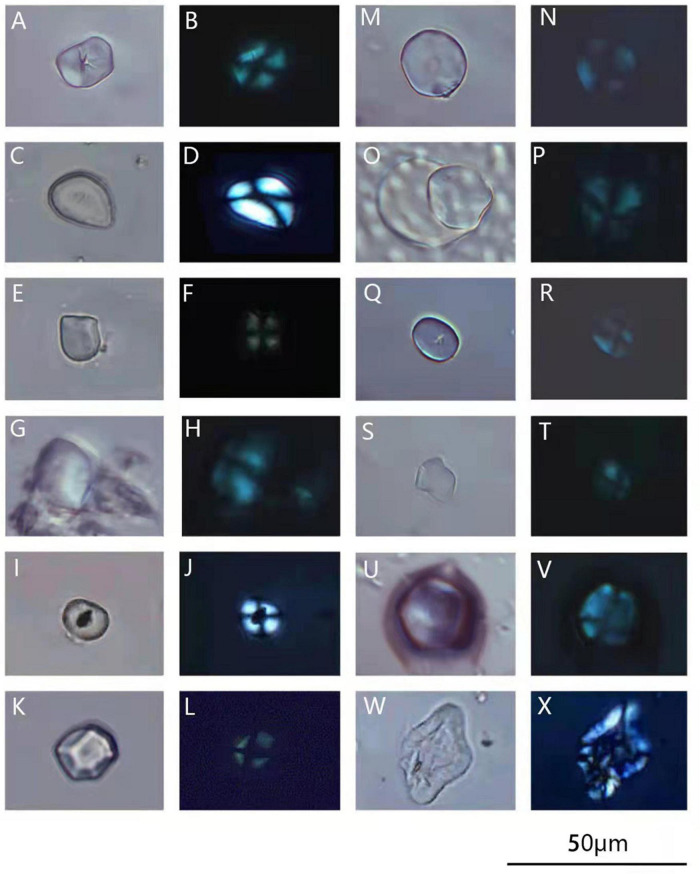
Microfossils of starch grains observed in the dental calculus of Fuyan Cave hominins teeth (each grain shown in unpolarized and polarized views). **(A–F)** Type 1, likely acorns; **(G–L)** type 2, possible tuber or root; **(M,N)** type 3, possible the tribe Triticeae; **(O,P)** type 4, possible Poaceae; **(Q–X)** starch grains lacked any known diagnostic features (Scale bar = 20μm).

Thirteen starch granules consistent with acorns represented this type. The starch grains from acorns have different morphologies according to their species and genera (Yang et al., 2009; Ge, 2010; Tao et al., 2011, 2015). Four starch granules of these were large (with size range 14.31–22.98 μm). As shown in Figures 3A,B, the granule was oblong with rounded corners and a centric hilum with visible fissures. The extinction arm bends with an “X” shape. The length of the major axis was 20.1 μm, and the minor axis was 15.3 μm. Our modern reference material (Supplementary Figures 3A,B) and previous study (Yang, 2017) all demonstrate that the extinction arm of acorns was bent or “Z” shaped, some with visible fissures. Their morphologies are different from those of other plants such as *Coix*, whose starch grains possessed the “Z” shaped extinction arm. According to the comparison of the size, shape, cross sections and other surface features, these starch granules matched acorns possibly from *Castanopsis* sp. The shape of the three starch grains is triangle with round corners (Figures 3C,D). The extinction was in the shape of “X.” The morphology of this type of starch grains is consistent with the characteristics of acorns based on modern starch references (Supplementary Figures 3C,D) and previous study (Yang et al., 2009; Tao et al., 2011). Starch grains from nuts, roots and tubers were semi-compound from previous study (Tao et al., 2011; Wang, 2017). The hilum of semi-compound (including bell shape) starch grains from tuber plants such as *Trichosanthes kirilowii*, *Colocasia*, *Dioscorea*, are almost eccentric and show a “X-shaped” extinction (Wang, 2017). However, the hilum of semi-compound starch grains from acorns are centric with “ + “ shape extinction (Yang et al., 2009). Six larger single starch grains with similar size and “ + “ shape extinction arms, which were also found based on modern references (Supplementary Figures 3D–H). These starch grains (Figures 3E,F) are likely consistent with acorns such as *Cyclobalanopsis* sp., *Castanopsis* sp. (Ge, 2010; Tao et al., 2011). In terms of morphology, this type of starch grain may represent acorns not tubers.

### Type 2

Four starch granules of this type were found. One starch grain was nearly oval with an eccentric hilum and showed faint extinction crosses under a polarized microscope (Figures 3G,H). This type of starch grain is possibly a root or tuber starch, as it exhibited an eccentric hilum (Wan et al., 2012; Wang, 2017). Three starch granules were polygonal with multiple extrusion surfaces. Under the polarized light the extinction arm was vertical (Figures 3K,L). On the basis of our modern reference (Supplementary Figures 2J–M) and previous studies (Wan et al., 2012; Wang, 2017), those are similar to modern starch grains from roots of *Pueraria lobata*, with characteristic pressure facets. Although it is difficult to identify these starches at the species level, the shapes suggest they might come from roots and tubers.

### Type 3

This group contained nearly round starch grains in which possessed an open and centric hilum, without any lamellae showing an extinction cross under the polarized light (Figures 3M,N). These starch granules were larger; the lengths of the A axis were 16 and 18 μm and those of the B axis were 15.1 and 17 μm. On the basis of published records (Piperno et al., 2004; Yang and Perry, 2013) and our modern reference (Supplementary Figures 3N–P), a bimodal size distribution of large and small granules with lenticular/discoidal or spherical in shape is one of the characteristics of cereal starches from the tribe Triticeae. Type 3 starch grains show the resemble morphologies with modern starch grains from the tribe Triticeae. We identify these grains belonging to the tribe Triticeae.

### Type 4

One polygonal starch grain had a centric hilum, without any lamellae showing an extinction cross under the polarized light. The length of the major axis was 15.3 μm and that of the minor axis was 15.2 μm (Figures 3O,P). Modern reference (Yang and Perry, 2013) revealed that starch grains with polygonal shapes usually come from the caryopsis of Poaceae. Due to the limited number of starch grain, this type is tentatively classified as Poaceae.

### Type 5

Twelve starch granules were included into this type because they are potentially diagnostic features. But they are not identified because they don’t match anything from our own and other published references (Figures 3I,J,Q–X). Four starch granules were defined by having a shared unique shape and probably represented a single plant taxon (Figures 3Q–T). Two more starch granules were sub-spherical and displayed a distinctive dark central vacuole (Figures 3I,J). The length of the major axis was about 11.3 μm, and that of the minor axis was about 8.6 μm. In addition, there were six starch granules (Figures 3U–X) that may have experienced processing damage based on previously published characteristics (Henry et al., 2009; Buckley et al., 2014).

**FIGURE 4:**
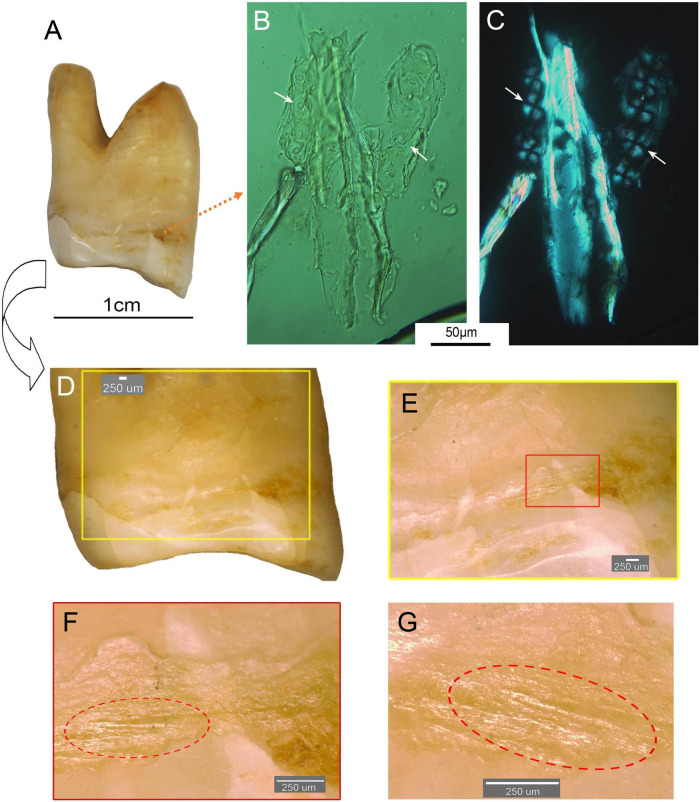
Wood fragments with characteristic conifer tracheid fibers in the dental calculus and parallel micro scratches in the groove of Fuyan Cave hominins teeth. **(A)** No. DX4 human teeth; **(B)** bordered pits of tracheid under unpolarized light from dental calculus; **(C)** bordered pits of tracheid and the “cross” feature under polarized light from dental calculus (Scale bar = 50 μm). **(D)** Parallel micro scratches in the groove of teeth, **(E–G)** parallel micro scratches in **(D)** at magnification 50×, 200× (Scale bar = 250 μm).

In addition, two wood fragments with characteristic conifer tracheid fibers were identified in the dental calculus of Fuyan Cave hominins teeth (Figure 4A). Identifying features included tubular cells with parallel lines of small, regular, circular bordered pits that often display a “cross” feature in cross polarized light (Figures 4B,C and Supplementary Figure 4). The identified features were very similar to those described in Hardy et al. (2012) and Radini et al. (2016), which support that this evidence is indeed preserved in dental calculus. In one of Fuyan Cave hominins teeth (No. DX4), a potential tooth-picking mark was found under the digital microscope (VHX-600) and showed a groove in the crown with numerous fine and parallel scratches (Figures 4D–G).

## Discussion

Despite the modest amount of starch grains found in the dental calculus samples and the identifications are presented as possibilities that may become secure identifications with further comparative work, the results revealed direct evidence that early modern human ate acorns, roots, tubers, grass seeds, and other yet-unidentified plants as food. The microremains we extracted from the samples did not find any clues to rice remains of Holocene agricultural population crops, but showed the typical characteristics of hunter-gatherers in Late Pleistocene.

Forty percent of the starch grains analyzed were likely from acorns. These findings show that acorns were potentially an abundant food resource. Nuts generally refer to the acorns of *Quercus, Lithocarpus, Castanopsis, Cyclobalanopsis*, and other plants of Fagaceae. Nuts today are widely distributed in China (Yang et al., 2009; Yang, 2017), including around Fuyan Cave. Exploitation of acorns as a carbohydrate source is well documented in prehistoric and recent times. Starch grains on grinding stones and pottery from numerous sites in China have been reported from the Upper Paleolithic to the Middle Neolithic (Piperno et al., 2000; Yang et al., 2009; Liu L. et al., 2010; Tao et al., 2011).

Our study provides the earliest evidence that acorns may have played an important role in human subsistence strategies approximately 80 ka in Late Pleistocene China. We also provide evidence that these plants have been exploited for a long time, and that humans started using this food resource earlier than previously estimated. Some species of acorns, such as *Castanopsis* sp. and *Cyclobalanopsis* sp., constitute a particularly valuable nutritional source (Preedy and Watson, 2020). These plants probably increased intake of vegetable fat and would likely have facilitated early modern humans’ access to food energy.

Modern humans also need a mixture of dietary carbohydrates to support the normal functioning of the brain, red blood cells and reproductive tissue (Wong et al., 2006; Hardy et al., 2015). Roots and tubers are rich in carbohydrates (Hardy et al., 2018). Therefore, roots and tubers, as important food sources, are an important factor in early human body evolution. However, roots and tubers are perishable and difficult to preserve, and it is difficult to obtain their macro remains in most cases. In contrast, starch grains extracted from dental calculus are good direct evidence of root and tuber consumption (Chandler-Ezell et al., 2006; Henry et al., 2012). Starch granules of roots and tubers have been reported from numerous sites dating from the Paleolithic to the Neolithic (Li et al., 2010; Wan et al., 2012; Wang, 2017; Zhang et al., 2017).

Dioscorea starches have been found from grinding stones at the Shizitan site, which dates back to 28–18 ka (Yang et al., 2015; Liu et al., 2018). Our evidence indicates that humans in South China ate carbohydrates from roots and tubers, at least from 80 ka. Our findings greatly advance our understanding of the utilization history of roots and tubers.

Triticeae plant remains were first found at the Shizitan site as far back as 28 ka (Yang et al., 2015). Two Triticeae starch grains were also recovered from the dental calculus of human teeth from Fuyan Cave in Hunan, South China. Although only two grains were recovered, this indicated that the use of Triticeae plants began at least 80 ka. In addition to Triticeae, starch grains from other unknown seeds might also be found.

Our results showed that the earliest direct evidence for human use of these wild grass seeds for food in the MIS5 period was approximately 80 ka, which indicates a long-lasting tradition of using Triticeae plants for subsistence during the Late Pleistocene in China. These findings also suggest a very long history of Triticeae exploitation as part of broad-spectrum subsistence strategies prior to its domestication.

High intake of starch plants (such as acorns) can lead to a certain degree of dental caries because frequent consumption of carbohydrates is a key factor in the initiation and progression of this disease (Humphrey et al., 2014). One early modern human in Daoxian had obvious dental caries (Figure 2C) and starch grains likely from acorns such as *Castanopsis* sp. were also found in this individual’s dental calculus (No. DX7). This occurrence of dental caries may have been related to the consumption of carbohydrates found in acorns.

Acorns including *Castanopsis* sp. and *Cyclobalanopsis* sp. have tannin and need to be processed before human consumption (Mason, 1996; Xiao et al., 2006; Liu et al., 2018). The earliest acorns associated with pitted stone tools have been found at the Early Middle Pleistocene in the Near East (Goren-Inbar et al., 2002), suggesting that acorns were indeed processed and then consumed, probably before modern humans. Unfortunately, no stone tools have been found in Fuyan Cave, and it was not possible to reveal whether the site includes food processing activities such as nut processing. It is worth mentioning that some starch grains displayed damage possibly caused by food processing. Food processing technology seems to have developed much earlier than previously detected, and early modern humans in Fuyan Cave may have had this technology 80 ka.

Coniferous fragments were found in the dental calculus of two Fuyan Cave hominins teeth 80 ka. Similar remains were also found in the dental calculus of a Neanderthal population from a 49,000-year-old site in Spain (Radini et al., 2016). Trace analysis evidence suggested that Neanderthals were accustomed to rubbing material between teeth (Radini et al., 2016). Interestingly, signs of similar behavior were also found in the teeth of Fuyan Cave hominins (No. DX4). A groove with numerous fine and parallel scratches was also found from the same tooth of its crown located at the buccal aspect of the mesial cervical line and above the mesial interproximal wear facet (Figure 4A). Coniferous fragments were found in the attached dental calculus of the teeth in which scratches were found. The coniferous fragments (Figures 4B,C) and parallel fine scratches in the groove (Figures 4D–G) indicate that Fuyan Cave hominins habitually rubbed material between their teeth, similar to Neanderthals. This is important evidence of tooth-picking behavior in early modern humans in East Asia.

Our results support that consumption of increased amounts of starch may have provided a substantial evolutionary advantage for early modern humans in the late Middle and early Upper Pleistocene in East Asia because of the energy it supplied to their increasingly large brain and other glucose-dependent tissues.

## Conclusion

We found acorns, roots and tubers, wild grass seeds, and other yet-unidentified starch grains from the dental calculus of Fuyan Cave hominins approximately 80 ka. This result reveals that these carbohydrates-rich foods played important roles in subsistence of the earliest modern humans in East Asia. Our study provides evidence of starch grains from acorns in dental calculus dating back at least 80 ka in South China. Additionally, starch grains that likely represent Triticeae were also found; although few were found, we should not underestimate the potential importance of wild grass foods as early as 80 ka in South China. Starch analysis of the earliest modern human dental calculus from Fuyan Cave documents definite consumption of plant food resources approximately 80 ka in the Late Pleistocene China. Furthermore, the discovery of different starch grains indicates that Fuyan Cave hominins may have ingested a variety of starchy plants to obtain carbohydrates and other energy to survive. Indeed, our study both reveals novel information about the diet of early modern humans in Late Pleistocene China and provides the earliest direct evidence for understanding the lifestyle of early modern humans in the late Middle and early Upper Pleistocene.

## Data availability statement

The original contributions presented in this study are included in the article/Supplementary material, further inquiries can be directed to the corresponding author.

## Author contributions

YW designed the research. YW, DT, XW, and WL performed the research and analyzed the data. YW and DT wrote the manuscript. All authors contributed to the article and approved the submitted version.
