# Supplementary material for: Diet of the earliest modern humans in East Asia
Source: Front Plant Sci. 2022 Aug 31;13:989308. doi: 10.3389/fpls.2022.989308 (PMC9471156; doi:10.3389/fpls.2022.989308)
Supplement: Supplementary file 1 [file Table_1.DOCX]

Figure S1. Several phytoliths extracted from surface sediments of *Stegodon orientalis* teeth from Fuyan Cave *(a)* rectangular, *(b)* bulliform, *(c)* elongate echinate, *(d)*smooth elongate.

Figure S2. Starch grains extracted from modern acorns, tubers and Triticeae in China (each grain shown in unpolarized and polarized views)*. (a-i)* modern acorns *(a-c) Castanopsis fargesii; (d-f) Cyclobalanopsis gilva; (g) Cyclobalanopsis glauca; (h) Cyclobalanopsis delavayi; (i) Cyclobalanopsis glauca; (j-m)* tubers, *Pueraria lobata; (n-p)* Triticeae *(n) Secale montanum; (0-p) Elymus sibiricus* (Scale bar= 50μm).

Figure S3. Modern reference plants commonly recovered from Neolithic sites in the middle Yangtze River area (each grain shown in unpolarized and polarized views) *(a) Oryza sativa; (b) Coix lacryma-jobi; (c) Nelumbo nucifera* (lotus root)*; (d-e) Nelumbo nucifera* (lotus fruit)*; (f) Euryale ferox; (g) Dioscorea polystachya; (h)* *Lithocarpus glabra (*Scale bar= 20μm).

Figure S4. Wood fragments with characteristic conifer tracheid fibers in the dental calculus of Daoxian human teeth. A and C, Bordered pits of tracheid under unpolarized light; B and D, bordered pits of tracheid and the ‘cross’ feature under polarized light (Scale bar= 50μm).
