# Supplementary material for: Diet of the earliest modern humans in East Asia
Source: Front Plant Sci. 2022 Aug 31;13:989308. doi: 10.3389/fpls.2022.989308 (PMC9471156; doi:10.3389/fpls.2022.989308)
Supplement: Supplementary file 2 [file Table_2.DOC]

**Table S1. List of starch grains and wood fragments observed in the dental calculus of Daoxian human teeth**

| **Sample No.** | **Specimens number** | **Teeth Type** | **Starch Grains** | | | | | | **Total** | **Wood fragments** |
| --- | --- | --- | --- | --- | --- | --- | --- | --- | --- | --- |
|  |  |  | **Type1 nuts** | **Type2**  **root or tuber** | **Type3**  **Triticeae** | **Type4**  **Poaceae** | **Type5**  **Unidentified** | **Type5**  **Damaged** |  |  |
| **DX1** | **PA1543** | **Left M^1^** |  | **1** | **1** |  | **1** |  | **3** |  |
| **DX2** | **PA1544** | **Left C^1^** | **1** | **2** |  |  | **1** | **1** | **5** |  |
| **DX4** | **PA1546** | **Left M^1^** |  |  |  |  |  |  | **0** | **1** |
| **DX5** | **PA1547** | **Right M^1^** |  |  |  | **1** |  |  | **1** |  |
| **DX6** | **PA1548** | **Right M^1^** |  |  |  |  | **1** |  | **1** |  |
| **DX7** | **PA1549** | **Right C^1^** | **3** |  |  |  |  |  | **3** |  |
| **DX8** | **PA1550** | **Left M^1^** |  |  |  |  |  | **1** | **1** |  |
| **DX9** | **PA1551** | **Left M_2_** |  |  |  |  |  |  | **0** | **1** |
| **DX11** | **PA1553** | **Left I_2_** | **1** |  |  |  | **1** |  | **2** |  |
| **DX13** | **PA1555** | **Left P3** | **2** |  |  |  |  | **1** | **3** |  |
| **DX18** | **PA1560** | **Right C_1_** |  | **1** |  |  |  |  | **1** |  |
| **DX19** | **PA1561** | **Left M_2_** | **1** |  | **1** |  |  | **1** | **3** |  |
| **DX23** | **PA1565** | **Left C_1_** | **2** |  |  |  |  | **2** | **4** |  |
| **DX25** | **PA1567** | **Right M_1_** | **2** |  |  |  | **2** |  | **4** |  |
| **DX27** | **PA1569** | **Right M_3_** | **1** |  |  |  |  |  | **1** |  |
|  |  |  | **13** | **4** | **2** | **1** | **7** | **5** | **32** | **2** |
