# Supplementary figures and images for: Diet of the earliest modern humans in East Asia
Source: Front Plant Sci. 2022 Aug 31;13:989308. doi: 10.3389/fpls.2022.989308 (PMC9471156; doi:10.3389/fpls.2022.989308)

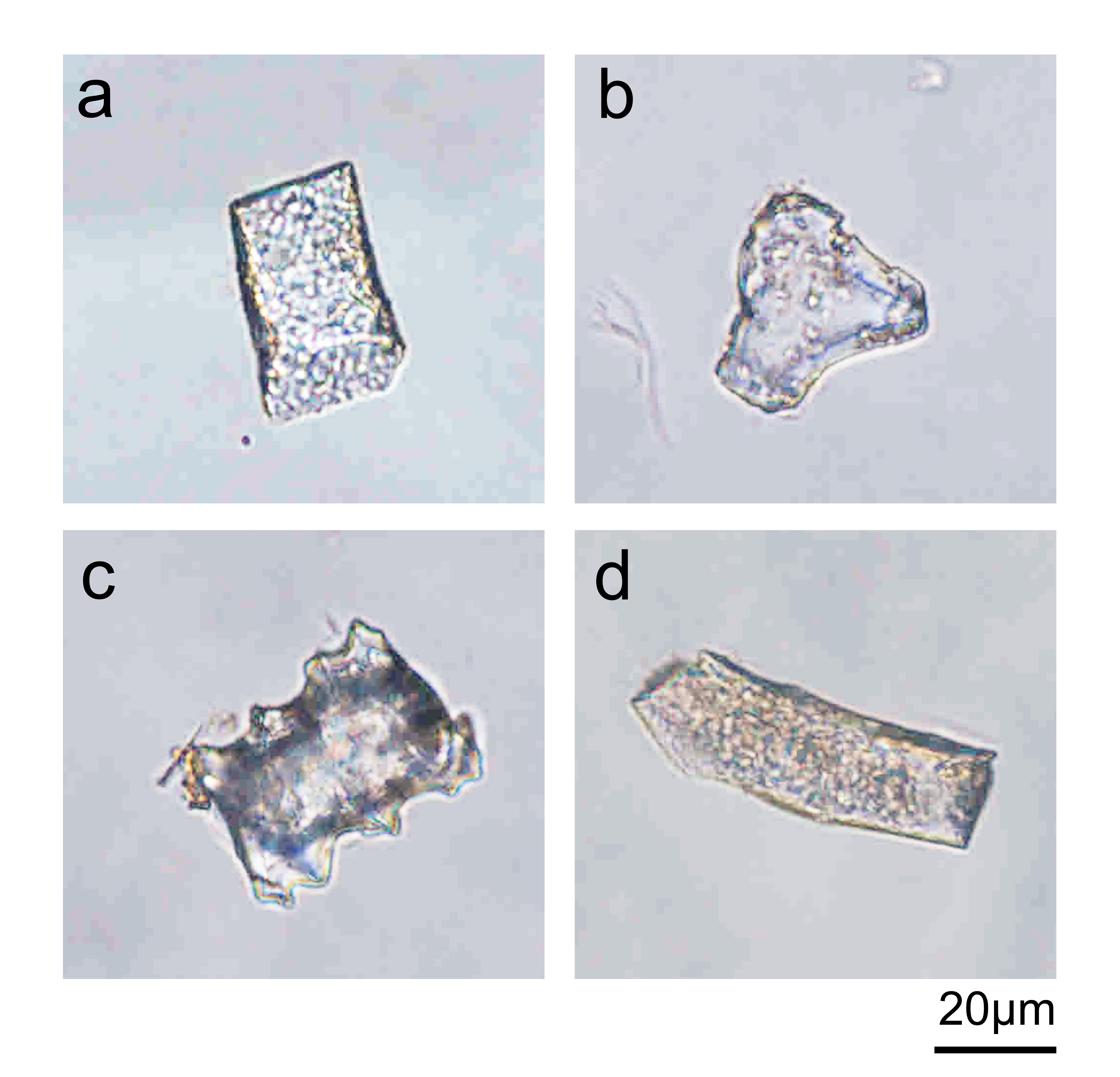

Supplement: Supplementary file 3 [file Image_1.JPEG]

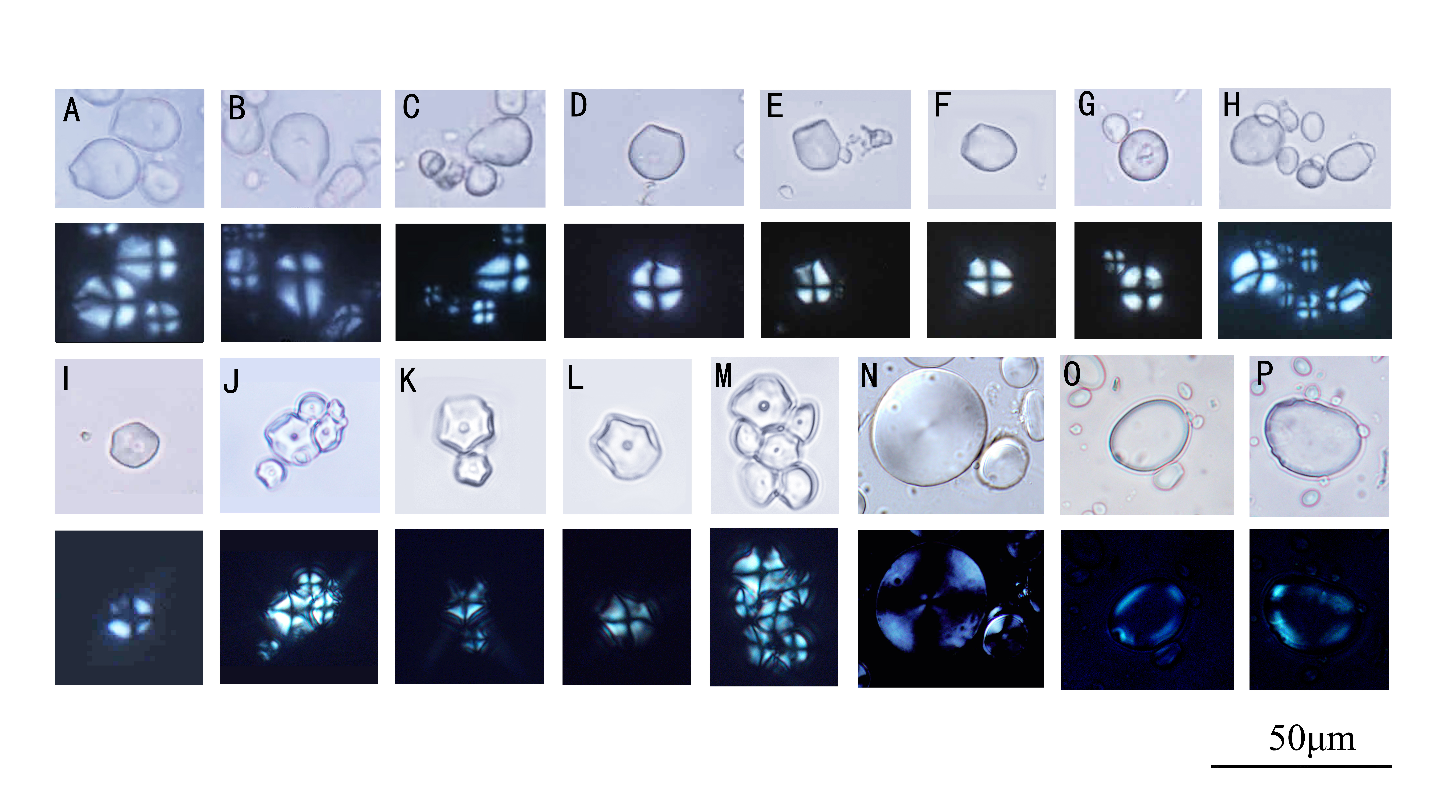

Supplement: Supplementary file 4 [file Image_2.JPEG]

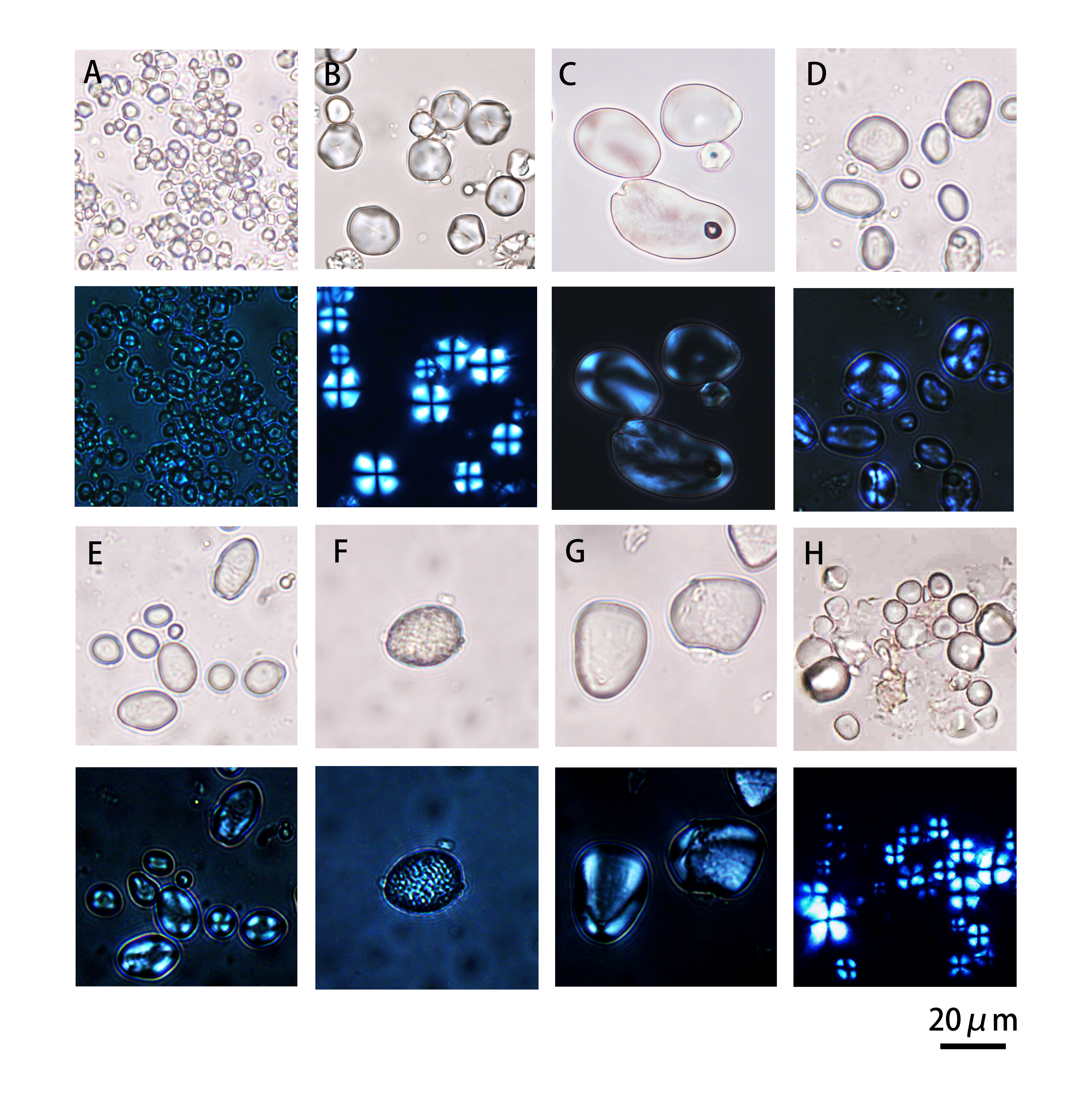

Supplement: Supplementary file 5 [file Image_3.JPEG]

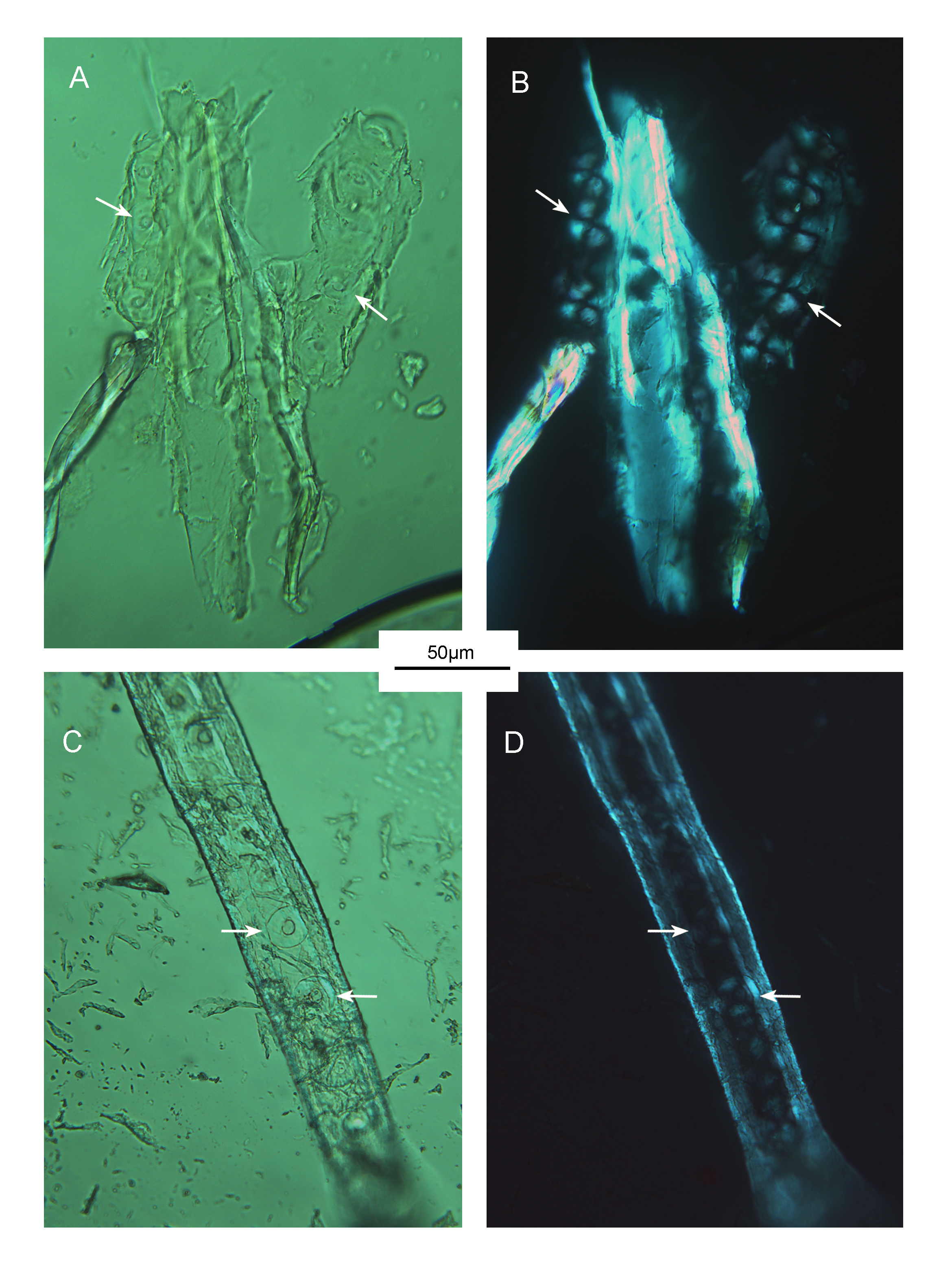

Supplement: Supplementary file 6 [file Image_4.JPEG]
